# A flexural exanthem following postexposure prophylaxis

**DOI:** 10.1111/ced.15131

**Published:** 2022-03-17

**Authors:** Darren Roche, Gregg Murray, Catriona Hackett, Anne‐Marie Tobin

**Affiliations:** ^1^ Department of Dermatology Tallaght University Hospital Dublin Ireland

## Abstract

We report a case of symmetrical drug‐related intertriginous and flexural exanthema following antiretroviral postexposure prophylactic medications, tenofovir and emtricitabine, commencement of which preceded the onset of the rash. Tenofovir and emtricitabine are both nucleoside reverse transcriptase inhibitor medications, commonly used to prevent development of AIDS.

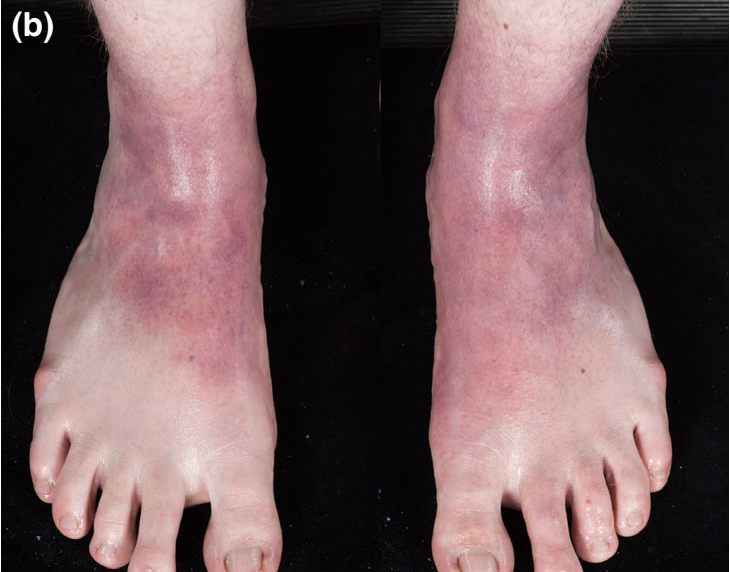

## Clinical findings

**Figure 1:**
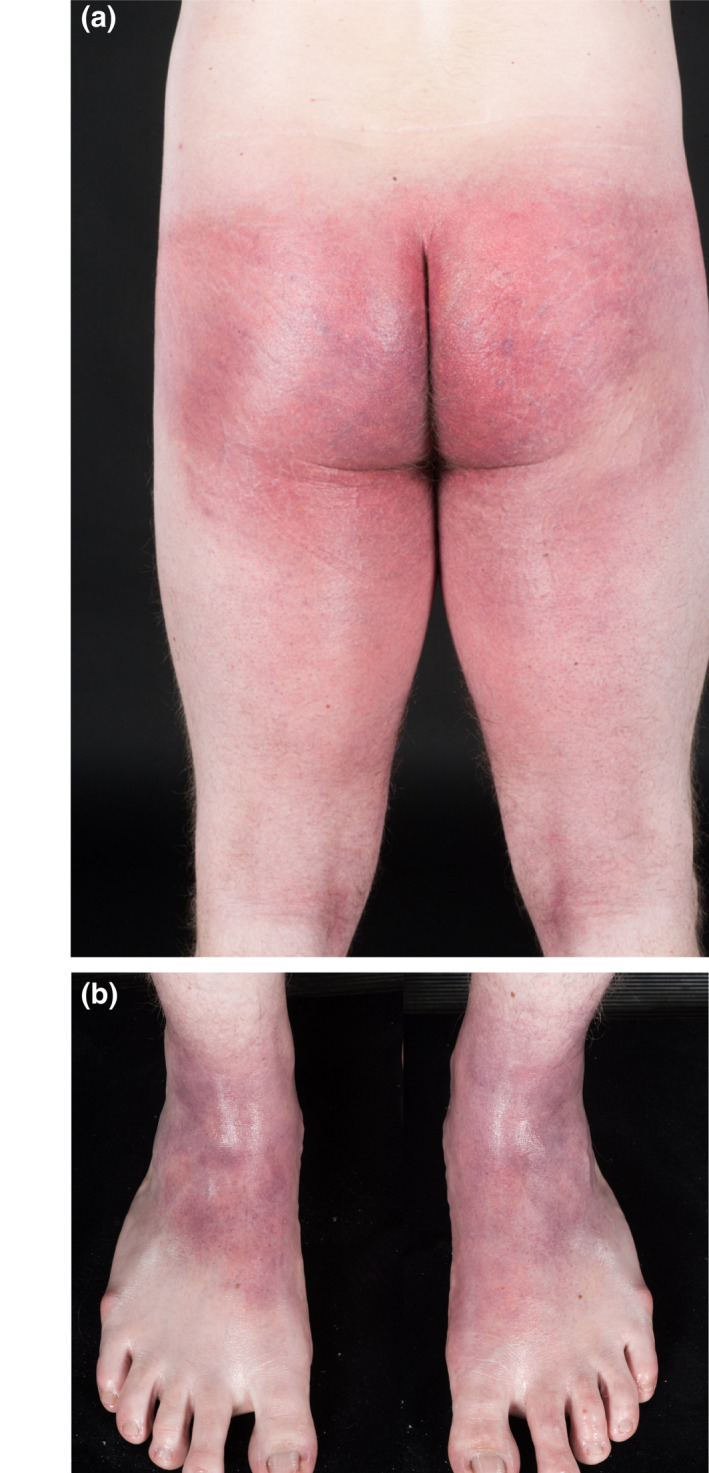
(a,b) Erythematous rash on (a) the legs and (b) feet.

A 26‐year‐old man presented as an emergency with a 24‐h history of a rapidly progressive, pruritic, painful rash. He had no relevant medical or dermatological history and took no regular medications. Two days previously, he had engaged in unprotected anal intercourse, and the following day, had attended a rapid‐access sexual health clinic. Following assessment, he was given postexposure prophylaxis (PEP) for HIV, comprising combination tenofovir and emtricitabine. Two hours following the first dose, the patient developed bilateral axillary pruritus. He had not taken any other medications or recreational drugs for 12 weeks prior to presentation, and had not taken PEP previously. He rapidly developed a widespread symmetrical erythematous tender rash, with a predilection for flexural sites (axillae/groin/buttocks/dorsal ankles) (Fig. 1a,b).

## Histopathological findings

**Figure 2:**
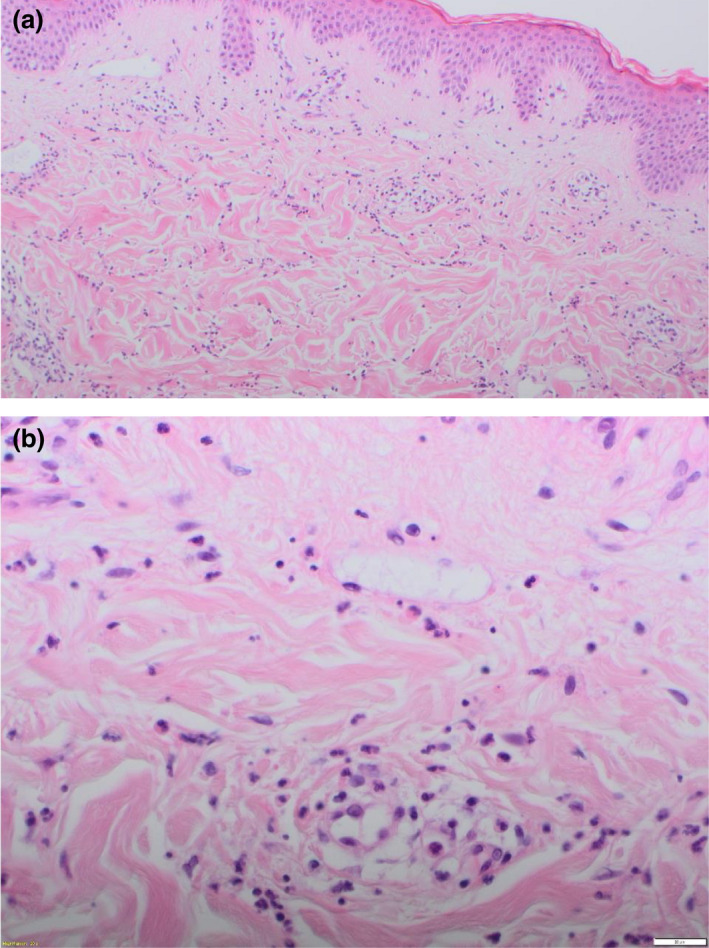
(a,b) Superficial perivascular and interstitial mixed inflammatory infiltrate, composed of lymphocytes, neutrophils and eosinophils. Haematoxylin and eosin, original magnification (a) × 10; (b) × 40.

Following initial review, a biopsy was taken from representative skin. Histopathological examination revealed a superficial perivascular and interstitial mixed inflammatory infiltrate, composed of lymphocytes, neutrophils and eosinophils (Fig. 2a,b). Epidermal changes included spongiosis.

What is your diagnosis?

## Diagnosis

Symmetrical drug‐related intertriginous and flexural exanthema (SDRIFE) secondary to PEP.

## Discussion

SDRIFE is an underdiagnosed drug eruption, characterized by sharply demarcated, flexural erythema without significant systemic symptoms.^1^ The older term ‘baboon syndrome’ has been deemed culturally inappropriate and too broad, as it originally referred to the resultant eruption from systemic exposure not only to medications but also to various contact allergens including metals, plants and herbals, and chemicals.^2^

A number of diagnostic criteria for SDRIFE have been proposed, including sharply demarcated, symmetrically distributed erythema of the gluteal/perianal/inguinal/perigenital area, following exposure to a systemic medication and absence of systemic symptoms and signs.^2^, ^3^, ^4^, ^5^ The primary morphology is variable, and can include plaques, papules or patches with or without scale, and colour varying from erythematous to dusky, hyperpigmented or violaceous.^6^ The latency between drug exposure and clinical manifestations in SDRIFE is also variable, from hours to days.^1^ The most commonly associated medications include aminopenicillins, β‐lactam antibacterials, chemotherapeutic agents, hydroxyzine and itraconazole. Tenofovir and emtricitabine are both nucleoside reverse‐transcriptase inhibitors. A wide range of cutaneous adverse effects for these drugs have been reported, including drug reaction with eosinophilia and systemic symptoms (DRESS), toxic epidermal necrolysis and leucocytoclastic vasculitis with tenofovir, and bullous, pustular, maculopapular and urticarial eruptions, pruritus and hyperpigmentation with emtricitabine. The combination therapy has been reported to cause drug‐induced lupus erythematosus. To our knowledge, there has been no previous case reported of SDRIFE in the literature.

A variety of histopathological appearances of SDRIFE has been reported. Commonly, a superficial perivascular infiltrate of mononuclear cells is observed.^6^ Epidermal changes such as spongiosis and acanthosis are also seen. Other reported features include subcorneal pustules, necrotic keratinocytes, and vacuolar changes and hydropic degeneration in the basal cell layer with subepidermal bullae. From a diagnostic perspective, consistent (rather than definitive) histological findings plus clinical diagnostic criteria are indicative of SDRIFE. The gold standard test in SDRIFE investigations to determine the causative agent is drug provocation testing; although patch testing and lymphocyte transformation testing have been reported as useful, they are not definitive, with patch test positivity reported as approximately 50%.^1^ Unfortunately, such testing could not be performed in our case, as the patient did not return for follow‐up; however, as our case fulfilled all the proposed clinical diagnostic criteria, we believed the diagnosis of SDRIFE is correct.

The differential diagnosis for SDRIFE includes common drug eruptions, fixed drug eruption (FDE), acute generalized exanthematous pustulosis (AGEP), DRESS, early morbilliform drug reactions and toxic erythema of chemotherapy, along with nondrug‐related causes such as systemic contact dermatitis due to allergens other than drugs, allergic and irritant contact dermatitis, seborrhoeic dermatitis, granular parakeratosis eruptions, eruptions with flexural predilection, intertrigo (caused by *Candida*, *Streptococcus*, *Staphylococcus* and erythrasma), tinea cruris, inverse/flexural psoriasis, pemphigus vegetans and Hailey–Hailey disease.^2^, ^5^ Once an underlying drug‐related cause is suspected, certain characteristics may differentiate SDRIFE from other conditions. With FDE, although it shares with SDRIFE a short latency between drug exposure and rash onset, and a lack of systemic symptoms, histological findings of localized, asymmetrical, round/oval lesions with a predominantly lymphocytic infiltrate favour FDE. AGEP may be distinguished from SDRIFE by a disseminated eruption comprised of nonfollicular pustules, often with systemic features such as fever and facial oedema, peripheral neutrophilia, and predominantly neutrophilic histology. DRESS may be distinguished from SDRIFE by systemic symptoms, other organ involvement and peripheral eosinophilia; however, histological differentiation from SDRIFE can be difficult and therefore, awareness of culprit medications, in combination with identification of suggestive clinical features and histological findings, is important in differentiating between SDRIFE and other common dermatoses.^2^, ^6^

In contrast to classic Type IV‐mediated delayed hypersensitivity reactions, SDRIFE has been reported to occur following exposure to a systemically administered drug at either the first or a repeat dose. SDRIFE may represent a distinct subgroup of Type IV drug eruption; however, theories relating to predisposing factors, such as local factors in flexures including increased density of apocrine/eccrine apparatus, friction or humidity, are unproven.

Treatment consists of removal of the causative agent where applicable, and treatment with topical corticosteroids. In our patient's case, both tenofovir and emtricitabine were discontinued, and the rash was treated with topical clobetasol ointment and paraffin gel four times daily. The rash improved over 5 days, with the patient remaining systemically well throughout. Investigations for other infectious causes, including SARS‐CoV‐2, were negative. Complete resolution of the rash occurred over the following weeks without complication.

The lack of defined pathogenic and immunological mechanisms and suggestive, rather than diagnostic, histological features indicate that correlation with proposed clinical criteria may be more useful in the diagnosis of SDRIFE. In our patient, commencement of PEP medications in the form of tenofovir and emtricitabine preceded the onset of a typical rash, fulfilling the proposed criteria for SDRIFE, and the histological investigation was consistent.
